# RNA-Seq analysis uncovers non-coding small RNA system of *Mycobacterium neoaurum* in the metabolism of sterols to accumulate steroid intermediates

**DOI:** 10.1186/s12934-016-0462-2

**Published:** 2016-04-25

**Authors:** Min Liu, Zhan-Tao Zhu, Xin-Yi Tao, Feng-Qing Wang, Dong-Zhi Wei

**Affiliations:** State key Lab of Bioreactor Engineering, Newworld Institute of Biotechnology, East China University of Science and Technology, Shanghai, 200237 China

**Keywords:** Noncoding RNA, Small regulatory RNA, RNA sequencing, Steroid catabolism, *Mycobacterium*

## Abstract

**Background:**

Understanding the metabolic mechanism of sterols to produce valuable steroid intermediates in mycobacterium by a noncoding small RNA (sRNA) view is still limited. In the work, RNA-seq was implemented to investigate the noncoding transcriptome of *Mycobacterium neoaurum (Mn)* in the transformation process of sterols to valuable steroid intermediates, including 9α-hydroxy-4-androstene-3,17-dione (9OHAD), 1,4-androstadiene-3,17-dione (ADD), and 22-hydroxy-23, 24-bisnorchola-1,4-dien-3-one (1,4-BNA).

**Results:**

A total of 263 sRNA candidates were predicted from the intergenic regions in *Mn*. Differential expression of sRNA candidates was explored in the wide type *Mn* with vs without sterol addition, and the steroid intermediate producing *Mn* strains vs wide type *Mn* with sterol addition, respectively. Generally, sRNA candidates were differentially expressed in various strains, but there were still some shared candidates with outstandingly upregulated or downregulated expression in these steroid producing strains. Accordingly, four regulatory networks were constructed to reveal the direct and/or indirect interactions between sRNA candidates and their target genes in four groups, including wide type *Mn* with vs without sterol addition, 9OHAD, ADD, and BNA producing strains vs wide type *Mn* with sterol addition, respectively. Based on these constructed networks, several highly focused sRNA candidates were discovered to be prevalent in the networks, which showed comprehensive regulatory roles in various cellular processes, including lipid transport and metabolism, amino acid transport and metabolism, signal transduction, cell envelope biosynthesis and ATP synthesis. To explore the functional role of sRNA candidates in *Mn* cells, we manipulated the overexpression of candidates 131 and 138 in strain *Mn*-9OHAD, which led to enhanced production of 9OHAD from 1.5- to 2.3-fold during 6 d’ fermentation and a slight effect on growth rate.

**Conclusions:**

This study revealed the complex and important regulatory roles of noncoding small RNAs in the metabolism of sterols to produce steroid intermediates in *Mn*, further analysis of which will promote the better understanding about the molecular metabolism of these sRNA candidates and open a broad range of opportunities in the field.

**Electronic supplementary material:**

The online version of this article (doi:10.1186/s12934-016-0462-2) contains supplementary material, which is available to authorized users.

## Background

Steroidal pharmaceuticals are of importance for the major challenges of contemporary society, such as life quality, healthy development, and aging, which are known to play a key role in the management of human fertility, osteoporosis, menopause, and blood pressure regulation. Steroid pharmaceuticals can rank among the most widely marketed category next to antibiotics and are important in the pharmaceutical industry. The annual production of steroid medications exceeds 1,000,000 tons and the global market is around US$ 10 billion [1–3].

Mycobacteria can use natural sterols as source of carbon and energy source, and interruptions in the catabolic pathway of sterols result in the accumulation of some key intermediates, such as 9α-hydroxy-4-androstene-3,17-dione (9OHAD), 1,4-androstadiene-3,17-dione (ADD), and 22-hydroxy-23, 24-bisnorchola-1,4-dien-3-one (1,4-BNA), which can be used as precursors to produce steroidal hormone pharmaceuticals [1, 4]. Dozens of steroid intermediates with varying biochemical properties can be obtained by the catabolism of sterols using mycobacteria [5]. It is a powerful tool for efficient production of active pharmaceutical ingredients and key intermediates from inexpensive sterol substrates by microbial steroid transformation [1]. In recent years, due to an excellent work conducted by van der Geize et al., many genes and pathways involved in sterol catabolism have been identified in *Rhodococcus* and *Mycobacterium* species [6]. Along with the advances in metabolic and genetic engineering, successes have been achieved in the pathway engineering to produce a plurality of valuable steroid intermediates in the nonpathogenetic and fast-growing mycobacteria, such as *M. neoaurum* (*Mn*) [4, 7, 8]. However, the current knowledge of global cellular metabolism to produce steroid intermediates is still limited, which greatly restricted the global metabolic regulation of mycobacterial cells to enhance their capacity and productivity for producing valuable steroid intermediates.

Generally, efforts to understand the molecular basis of gene regulation in mycobacteria are dominated by a protein-centric view. In recent years, bacterial small noncoding regulatory RNA (sRNA), typically 50–500 nucleotides in length, has proven to be potent and important regulators that plays a role in a wide variety of molecular mechanisms and is much more widespread than previously thought in prokaryotic organisms [9, 10]. In fact, there is evidence that regulatory RNAs represent more advantages than proteins for controlling gene expression, including: a lower energy cost for generating regulatory and target RNAs, much faster for controlling gene expression, and a rapid clearance efficiency because of less stable structures [9]. In many bacteria, the intergenic sequences have been demonstrated to contain sequences that encode small regulatory RNAs, which play an important role in the physiology of mycobacteria despite the mechanism of their action remains unclearly [11]. Typically, the majority of sRNAs regulate gene expression post-transcriptionally through base pairing with one or more target mRNAs, thereby altering translation efficiency, mRNA stability and transcription [12].

In mycobacteria, RNA regulators are frequently implicated in adaptive responses to environmental changes, such as temperature and pH, and metabolism of energy, lipids, and metabolites [13], and may therefore be expected to play a role in the sterol catabolism and steroid accumulation in *Mn*. The growing interest for gene regulation in mycobacteria has promoted the research of sRNAs. At the early stage, experimental approach was used for searching and characterizing regulatory sRNAs, such as the first complete experimental confirmation of sRNAs in *Mycobacterium tuberculosis* (*Mtb*) H37Rv by cloning and sequencing a fraction of shortRNAs [14] and using a combination of computer prediction and cloning approaches in *M. bovis* BCG [15]. In recent years, bacterial high throughput sequencing has emerged as a powerful tool for studying the noncoding sRNAs in mycobacteria. Several reports have been published with regards to the identification of sRNA candidates, including intergenic sRNAs and antisense RNAs, using sequencing combined with information analysis [11, 16, 17]. Investigations of mycobacterial non-coding sRNAs have mainly focused on *Mtb*. A number of reports have described the identification of several sRNAs that are likely to be responsible for adaptation and virulence in *Mtb*, such as F6, B55, B11, MTS2823, and mcr11 [14–16]. Nevertheless, to our limited knowledge, less attention has been given to the potential role of sRNAs in the metabolism of sterols in mycobacteria [12]. The regulatory mechanisms underlying these adaptations are largely unknown, prompting us to fill this gap in our knowledge.

To deepen the understanding of regulatory mechanism of sRNA for sterol catabolism and steroid production, we used transcriptomics to characterize the complex behavior of sRNAs necessary for the production of key steroid intermediates in mutant *Mn* cells, including 9OHAD, ADD, and 1,4-BNA. Totally, the noncoding transcriptome of five *Mn* samples were investigated, including wide-type *Mn* ATCC25795 cultured without (*Mn*-C) and with sterol addition (*Mn*-CC), mutant *Mn* ATCC25795 strains producing 9OHAD (*Mn*-9OHAD), ADD (*Mn*-ADD), and 1,4-BNA (*Mn*-BNA) with sterol addition. The differential expression of sRNA candidates in these *Mn* strains has been explored. Additionally, regulatory networks were constructed to reveal direct and/or indirect interactions between sRNA candidates and their target genes in various aspects of cellular process, including sterol catabolism, central carbon metabolism (CCM), transport, cell envelope biosynthesis, and ATP synthesis.

## Results

### In silico prediction of sRNA candidates in *Mn* cells

**Fig. 1:**
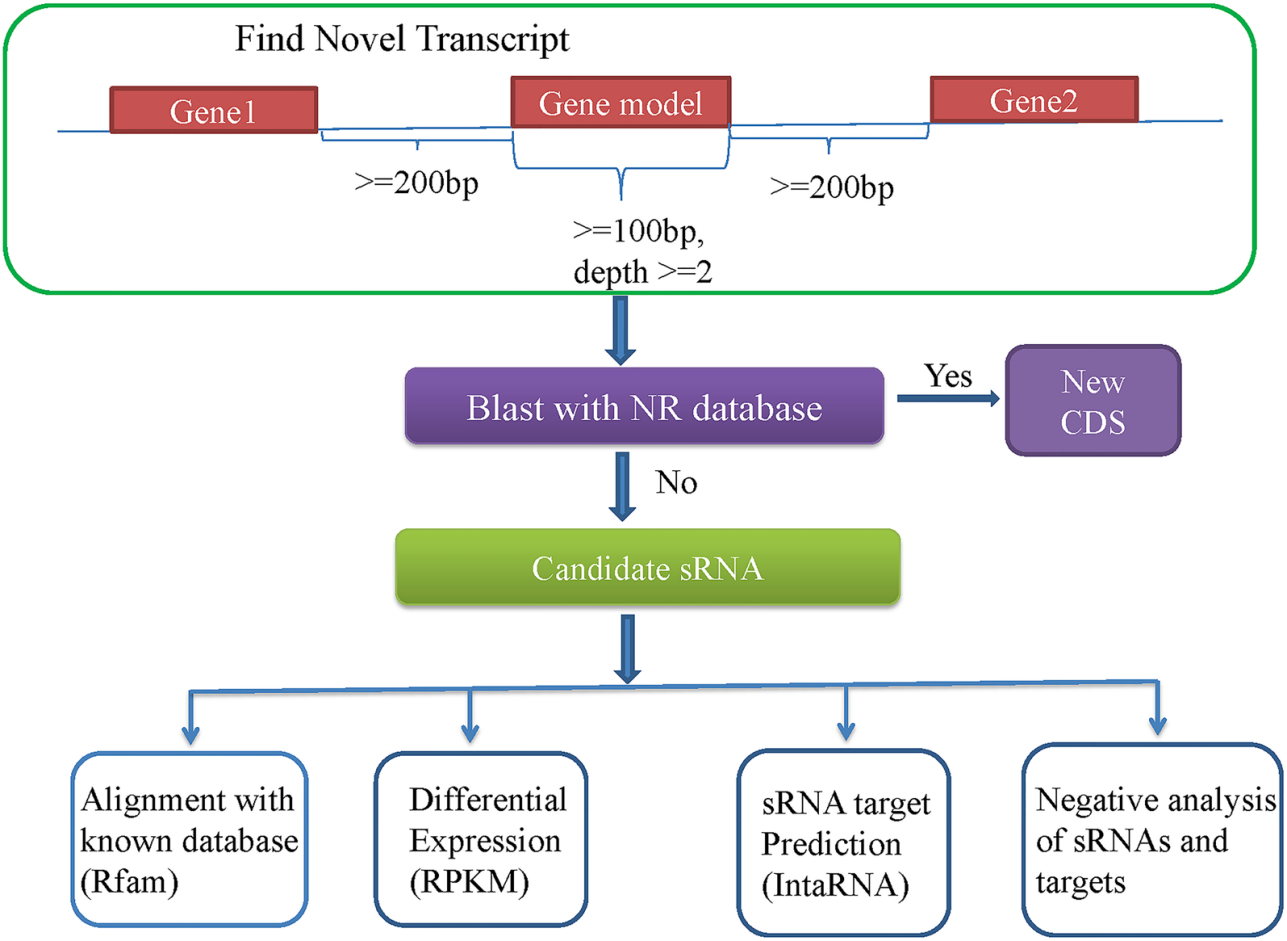
Outline of sRNA analysis

Novel transcripts can be found by high throughput sequencing since the present databases is incomplete for *Mn*. Usually, intergenic regions (200 bp away from upstream or downstream genes) were thought to be the possible source of novel transcripts. Based on RNA sequencing data, some novel transcripts from intergenic regions in strains *Mn*-C, *Mn*-CC, *Mn*-9OHAD, *Mn*-ADD, and *Mn*-BNA were predicted as shown in Fig. 1, and the results are shown in Additional file 1: Table S1. The novel transcripts were blasted against the non-redundant (NR) databases, and these could not be blasted against the NR databases were thought to be sRNA candidates. The sRNA candidates in *Mn* were predicted and analyzed using the strategy as described in Fig. 1. In total, 263 sRNA candidates were identified in this work (130 strand +; 133 strand −), and the chromosome location, length, sequences, secondary structure, and annotation results of these sRNAs are shown in Additional file 2: Table S2. Length distribution of sRNA candidates is shown in Additional file 3: Figure S1. The length of 263 sRNA candidates is ranged from 200 to 500 bp.

### Differential expression of sRNA candidates in various Mn strains

All mapped reads, except those mapping to the rRNA operon, were chosen for the total number of reads. Final RPKM values of sRNA candidates for each sample of *Mn*-C, *Mn*-CC, *Mn*-9OHAD, *Mn*-ADD, and *Mn*-BNA, together with comparative results in groups *Mn*-CC/C, *Mn*-9OHAD/CC, *Mn*-ADD/CC and *Mn*-BNA/CC, are shown in supplementary datasets Additional file 4: Table S3 (the entire list of differentially expressed sRNA candidates, including their RPKM levels, fold changes, P values and FDRs). Interestingly, multiple differentially expressed sRNAs were detected in the comparable groups of *Mn*-CC/C, *Mn*-9OHAD/CC, *Mn*-ADD/CC and *Mn*-BNA/CC. In *Mn*-CC/C, a total of 144 differentially expressed sRNA candidates were found, including 112 upregulated sRNAs and 32 downregulated sRNAs. The most abundant differentially expressed sRNAs in *Mn*-CC/C are shown in Table 1. In *Mn*-9OHAD/CC, *Mn*-ADD/CC and *Mn*-BNA/CC, 138, 122, and 129 differentially expressed sRNA candidates were found, including 50, 47, and 52 upregulated sRNAs and 88, 75, and 77 downregulated sRNAs, respectively. The most abundant upregulated and downregulated sRNA candidates in *Mn*-9OHAD/CC, *Mn*-ADD/CC and *Mn*-BNA/CC groups are shown in Tables 2 and 3, respectively. There are several shared upregulated sRNA candidates in *Mn*-9OHAD/CC, *Mn*-ADD/CC and *Mn*-BNA/CC groups, such as candidates 112, 105, 107, 188, 125, 255, 131, 138, 31, 25, 239, and 203. Meanwhile, there are also several shared downregulated sRNA candidates in *Mn*-9OHAD/CC, *Mn*-ADD/CC and *Mn*-BNA/CC groups, such as candidates 160, 209, 174, 13, and 250.

**Table 1:** Ranking of the most abundant differentially expressed sRNAs in *Mn*-CC/C

Upregulation				Downregulation	
SRNA ID	Fold change^a^	SRNA ID	Fold change	SRNA ID	Fold change
160	Infinity^b^	202	7.2	105	0
170	Infinity	53	6.6	107	0
130	Infinity	46	6.6	147	0
121	Infinity	246	6.1	188	0
151	Infinity	249	6.1	255	0
198	Infinity	213	6.1	248	0
257	Infinity	218	5.5	139	0
36	Infinity	94	5.5	131	0
233	Infinity	120	5.5	125	0
63	Infinity	67	5.5	138	0.14
98	Infinity	77	5.0	79	0.14
68	Infinity	3	5.0	234	0.17
48	Infinity	76	5.0	31	0.18
254	Infinity	169	5.0	203	0.22
83	Infinity	6	5.0	60	0.22
100	Infinity	108	4.9	129	0.28
42	Infinity	235	4.8	32	0.28
69	Infinity	157	4.8	75	0.28
222	Infinity	5	4.6	133	0.28
219	Infinity	86	4.4	24	0.28
51	Infinity	142	4.4	137	0.28
109	12.1	162	4.4	220	0.32
197	11.0	40	4.4	134	0.37
243	11.0	17	4.4	20	0.37
127	8.8	179	4.4	106	0.37
54	8.8	89	4.2	206	0.41
122	8.5	116	4.1	49	0.42

^a^ The RPKM ratio of sRNA candidate expressed in the form of *Mn*-CC/C

^b^ The RPKM of sRNA candidate in *Mn*-C is zero

**Table 2:** Ranking of the most abundant upregulated sRNAs in *Mn*-9OHAD/CC, *Mn*-ADD/CC, and *Mn*-BNA/CC

*Mn*-9OHAD/CC		*Mn*-ADD/CC		*Mn*-BNA/CC	
SRNA ID	Fold change^a^	SRNA ID	Fold change	SRNA ID	Fold change
112	Infinity^b^	147	Infinity	255	Infinity
105	Infinity	131	Infinity	112	Infinity
107	Infinity	248	Infinity	131	Infinity
188	Infinity	255	Infinity	139	Infinity
125	Infinity	139	Infinity	105	Infinity
255	Infinity	112	Infinity	248	Infinity
248	Infinity	105	Infinity	107	Infinity
131	Infinity	107	Infinity	147	Infinity
25	16.1	125	Infinity	125	Infinity
137	6.9	188	Infinity	188	Infinity
219	6.3	138	14.3	31	13.5
24	6.3	222	8.7	222	12.9
31	5.8	219	7.5	137	11.6
79	5.8	25	7.5	138	10.4
239	5.2	79	7.5	25	9.8
220	5.2	31	7.5	203	8.0
176	5.2	239	7.1	239	5.8
32	4.6	203	6.0	24	5.2
106	4.6	38	4.9	211	4.6
138	4.6	84	4.5	183	4.5
203	4.6	217	4.5	38	4.3
148	4.2	32	4.1	20	3.7
146	4.0	59	3.8	79	3.7

^a^ The RPKM ratio of sRNA candidate expressed in the form of *Mn*-9OHAD/CC, *Mn*-ADD/CC, and *Mn*-BNA/CC

^b^ The RPKM of sRNA candidate in *Mn*-CC is zero

**Table 3:** Ranking of the most abundant downregulated sRNAs in *Mn*-9OHAD/CC, *Mn*-ADD/CC, and *Mn*-BNA/CC

*Mn*-9OHAD/CC		*Mn*-ADD/CC		*Mn*-BNA/CC	
SRNA ID	Fold change^a^	SRNA ID	Fold change	SRNA ID	Fold change
160	0	160	0	160	0
102	0	47	0	37	0
184	0	88	0	236	0
8	0	60	0	60	0
111	0	221	0	42	0
225	0	223	0	8	0.10
151	0	75	0	121	0.10
165	0	65	0.04	127	0.11
33	0	210	0.07	94	0.12
233	0	209	0.08	65	0.12
187	0	172	0.11	108	0.14
17	0	46	0.13	142	0.15
73	0	174	0.13	196	0.15
57	0	101	0.13	260	0.15
100	0	27	0.14	151	0.15
75	0	167	0.14	152	0.16
108	0.05	91	0.15	122	0.17
209	0.06	243	0.15	115	0.18
178	0.07	127	0.16	235	0.19
10	0.08	225	0.17	174	0.20
190	0.11	14	0.19	85	0.20
128	0.12	207	0.19	111	0.20
130	0.14	246	0.20	209	0.20
116	0.15	166	0.20	9	0.20
114	0.17	164	0.20	164	0.21
13	0.16	41	0.21	205	0.21
250	0.16	13	0.21	13	0.22
170	0.19	190	0.21	228	0.22
121	0.19	149	0.21	250	0.22
129	0.19	250	0.21	70	0.22
168	0.19	235	0.23	210	0.23
246	0.20	122	0.25	120	0.24
152	0.22	70	0.26	97	0.24
192	0.23	89	0.26	14	0.24
260	0.23	39	0.27	88	0.24
47	0.23	161	0.28	89	0.24
67	0.23	120	0.30	41	0.26
166	0.24	97	0.30	172	0.26
174	0.24	67	0.30	198	0.26

^a^ The RPKM ratio of sRNA candidate expressed in the form of *Mn*-9OHAD/CC, *Mn*-ADD/CC, and *Mn*-BNA/CC

### Regulatory networks of sRNA candidates and their target genes

The functional characterization of sRNA candidates is challenging, which usually requires highly focused and extensive experimental procedures. Herein, we have predicted functional roles and regulatory interactions for differentially expressed sRNA candidates in *Mn* using a network biology approach. To identify the sRNA candidates involved in the regulation of sterol catabolism and intermediate accumulation, negative analysis for sRNAs and their target mRNAs was conducted. This was performed by using these differentially expressed sRNAs as well as a set of their significant predicted target genes involved in responses to environmental stresses for resolving a sRNA-target network. An important issue in sRNA biology is to identify the genes that these molecules regulate. Firstly, we have predicted the target genes of differentially expressed sRNAs in groups *Mn*-CC/C, *Mn*-9OHAD/CC, *Mn*-ADD/CC and *Mn*-BNA/CC using IntaRNA. The target mRNAs of differentially expressed sRNAs are shown in Additional file 5: Table S4. Subsequently, the sRNA-target networks for *Mn*-CC/C, *Mn*-9OHAD/CC, *Mn*-ADD/CC and *Mn*-BNA/CC were generated by connecting gene nodes and differentially expressed sRNA candidates using software Cytoscape. These four networks are shown in Additional file 6: Figure S2 and the details are shown in Additional file 7: Table S5.

To further explore the functional roles of sRNAs in *Mn*, we have analyzed the most abundant upregulated (Table 4) and downregulated (Table 5) sRNAs with focused roles in the regulatory networks of *Mn*-9OHAD/CC, *Mn*-ADD/CC and *Mn*-BNA/CC. For upregulated sRNA candidates, five highly focused sRNAs (defined as sRNAs with more than 10 target genes in the network) were found to be prevalently existed in these three groups, including 131, 138, 222, 79, and 112 (Table 4). For downregulated sRNA candidates, nine highly focused sRNAs were found to be prevalently existed in groups *Mn*-9OHAD/CC, *Mn*-ADD/CC and *Mn*-BNA/CC, including 225, 209, 122, 210, 166, 152, 164, 70, and 97 (Table 5).

**Table 4:** Representation of the most abundant upregulated sRNAs in regulatory networks of *Mn*-9OHAD/CC, *Mn*-ADD/CC, and *Mn*-BNA/CC

SRNA ID	*Mn*-9OHAD/CC		*Mn*-ADD/CC		*Mn*-BNA/CC	
	Fold change^a^	Num^b^	Fold change	Num	Fold change	Num
131	Infinity	14	Infinity	18	Infinity	15
138	4.6	13	14.3	21	10.4	18
222	2.3	27	8.7	40	12.9	32
79	5.8	17	7.5	27	3.7	22
112	Infinity^c^	18	Infinity	15	Infinity	19
255	Infinity	6	Infinity	13	Infinity	10
125	Infinity	4	Infinity	5	Infinity	5
188	Infinity	4	Infinity	9	Infinity	6
105	Infinity	5	Infinity	13	Infinity	10
107	Infinity	3	Infinity	9	Infinity	4
248	Infinity	1	Infinity	2	Infinity	2
146	4.0	33	3.8	52	NF	NF
66	2.3	11	2.4	18	NF	NF
20	NF^d^	NF	3.0	36	3.7	27
38	NF	NF	4.9	24	4.3	34

^a^ The RPKM ratio of sRNA candidate expressed in the form of *Mn*-9OHAD/CC, *Mn*-ADD/CC, and *Mn*-BNA/CC

^b^ Number of genes regulated by sRNA candidates

^c^ The RPKM of sRNA candidate in *Mn*-CC is zero

^d^ Not found

**Table 5:** Representation of the most abundant downregulated sRNAs in regulatory networks of *Mn*-9OHAD/CC, *Mn*-ADD/CC, and *Mn*-BNA/CC

SRNA ID	*Mn*-9OHAD/CC		*Mn*-ADD/CC		*Mn*-BNA/CC	
	Fold change^a^	Num^b^	Fold change	Num	Fold change	Num
225	0	57	0.17	50	0.40	48
209	0.06	64	0.08	43	0.20	48
122	0.42	30	0.25	24	0.17	34
210	0.47	38	0.07	34	0.23	40
174	0.24	18	0.13	9	0.20	16
166	0.24	21	0.20	17	0.42	17
152	0.22	31	0.49	26	0.16	29
246	0.21	1	0.21	2	0.31	1
164	0.26	16	0.21	14	0.21	15
170	0.19	12	0.44	3	0.36	5
70	0.44	27	0.26	25	0.22	23
67	0.23	11	0.30	8	0.40	7
97	0.46	16	0.30	18	0.24	21
89	0.45	7	0.26	7	0.24	11
121	0.19	6	0.38	6	0.10	8
181	0.33	36	0.38	29	NF	NF
3	NF^c^	NF	0.33	35	0.41	44
10	0.08	65	NF	NF	0.48	52
64	NF	NF	0.31	35	0.33	39
182	0.31	24	0.41	15	NF	NF
126	0.29	43	0.44	31	NF	NF
167	0.42	20	0.14	18	NF	NF
101	0.38	28	0.13	17	NF	NF
116	0.15	28	NF	NF	0.33	22

^a^ The RPKM ratio of sRNA candidate expressed in the form of *Mn*-9OHAD/CC, *Mn*-ADD/CC, and *Mn*-BNA/CC

^b^ Number of genes regulated by sRNA candidates

^c^ Not found

### In silico identification of pathways regulated by sRNA candidates

To understand the function of these sRNA candidates in cells, their target metabolism pathways were investigated. An in silico analysis was performed to identify the pathways regulated by these sRNA candidates. Using functional enrichment analysis, the molecular functions and pathways enriched in the sRNA-targeted genes were identified in four comparable groups of *Mn*-CC/C, *Mn*-9OHAD/CC, *Mn*-ADD/CC and *Mn*-BNA/CC. In the results, amino acid transport and metabolism (COG: E), inorganic ion transport and metabolism (COG: P), carbohydrate transport and metabolism (COG: G), cell wall/membrane/envelope biogenesis (COG: M), lipid transport and metabolism (COG: I), and secondary metabolites biosynthesis, transport and catabolism (COG: Q) were emerged as function potentially subjected to the regulation of sRNA candidates. The functional enrichment results in *Mn*-CC/C, *Mn*-9OHAD/CC, *Mn*-ADD/CC and *Mn*-BNA/CC and the corresponding targeted genes can be found in Additional file 8: Table S6.

### sRNA candidates directly associated with sterol catabolism

Usually, sterol catabolism by mycobacteria involves three major processes, i.e. the uptake of sterol, the oxidation of steroid nucleus, and the elimination of aliphatic side-chain at C17, the most genes responsible for which are gathered together as a gene cluster [6, 18]. To explore whether there are sRNAs directly associated with the metabolism of sterols, we identified some possible sRNA candidates (Table 6; Additional file 9: Table S7) responsible for sterol catabolism from the details of regulatory networks of sRNA candidates and their target genes (Additional file 7: Table S5). Overall, the sRNA candidates associated with sterol catabolism were different in four groups of *Mn*-CC/C, *Mn*-9OHAD/CC, *Mn*-ADD/CC and *Mn*-BNA/CC. However, there were still some shared sRNA candidates showing significant correlation with sterol catabolism in different groups (Table 6). For example, the sRNA candidate 17 associated with *kstR2*, a TetR-type transcriptional regulator regulating the expression of genes for rings C/D degradation, were found to be shared in groups *Mn*-CC/C and *Mn*-9OHAD/CC. Candidate 134 in *Mn*-CC/C, candidates 109, 209, and 205 in *Mn*-9OHAD/CC, candidates 109 and 209 in *Mn*-ADD/CC, and candidates 209 and 205 in *Mn*-BNA/CC are related to the same gene *mce4A*, one of the genes in *mce4* operon responsible for sterol transport. In addition, the sRNA candidates related with another gene *mce4F* in *mce4* operon are candidate 188 in *Mn*-CC/C, candidates 226 and 10 in *Mn*-9OHAD/CC, candidate 119 in *Mn*-ADD/CC, and candidates 226 and 10 in *Mn*-BNA/CC. These sRNA candidates, directly associated with sterol catabolism, are generally different but have shared features, especially in 9OHAD, ADD, and BNA producing strains. Many highly focused sRNA candidates in the regulatory networks are associated with the genes for sterol catabolism, such as candidate 138 for genes *ltp3*, *fadE26*, *fadE27*, and *fadE32*, candidate 131 for gene *fadA5*, candidate 79 for genes *hsaD*, *fadA5*, and *hsd4B*, candidate 209 for genes *mce4A*, *mce4D*, and *yrb4A*, candidate 225 for genes *yrb4A* and *ltp4*, and candidate 210 for genes *rv3541c* and *rv3542c* (Table 6 and Additional file 9: Table S7).

**Table 6:** The sRNA candidates related with genes for sterol catabolism

*Mn*-CC/C		*Mn*-9OHAD/CC		*Mn*-ADD/CC		*Mn*-BNA/CC	
Gene	SRNA ID	Gene	SRNA ID	Gene	SRNA ID	Gene	SRNA ID
*yrb4A*	234	*mce4A*	109, 209, 205	*mce4A*	109, 209	*yrb4A*	55, 225, 198, 14, 169, 209, 148
*mce4A*	134	*mce4B*	39	*mce4B*	235, 39	*yrb4B*	90
*mce4C*	253	*mce4C*	167	*mce4C*	167, 208	*mce4A*	209, 205
*mce4F*	188	*mce4D*	39, 116, 167, 215, 209	*mce4D*	39, 167, 215, 209	*mce4B*	235
*kshB*	188, 20	*mce4E*	102	*mce4E*	119	*mce4D*	116, 215, 209
*hsaD*	79	*mce4F*	226, 10	*mce4F*	119	*mce4E*	102
*hsaC*	206	*hsaF*	217	*kshA*	146	*mce4F*	226, 10
*ltp3*	138	*cyp142*	231, 126	*ltp3*	181	*cyp142*	42, 61
*fadD17*	49, 129	*aspB*	168	*ltp4*	225	*cyp125*	34
*fadE34*	137, 206	*hsd4B*	222, 79	*echA19*	235	*kshA*	180
*fadE26*	138, 20	*kstR2*	17	*fadD17*	97, 164	*hsaB*	116, 153
*fadE27*	138	–	–	*fadE32*	138, 66, 146	*ltp2*	195
*fadE28*	49, 60	–	–	*fadE28*	41, 126, 8, 190, 14, 97, 67, 60	*ltp4*	225
*fadE29*	129	–	–	*rv3542c*	210	*echA19*	235
*fadE30*	23	–	–	*rv3541c*	210	*fadD17*	49, 97, 164, 145
*fadA6*	43, 63	–	–	–	–	*hsd4A*	180
*fadA5*	131, 79	–	–	–	–	*fadE28*	41, 49, 1, 8, 190, 14, 97, 67, 60
*hsd4B*	79	–	–	–	–	*fadE32*	138
*kstR2*	17	–	–	–	–	*rv3542c*	34, 210
–	–	–	–	–	–	*rv3541c*	210

### sRNA candidates associated with basic metabolism, cell envelope biosynthesis, efflux pumps, and ATP synthesis in Mn-9OHAD/CC

**Fig. 2:**
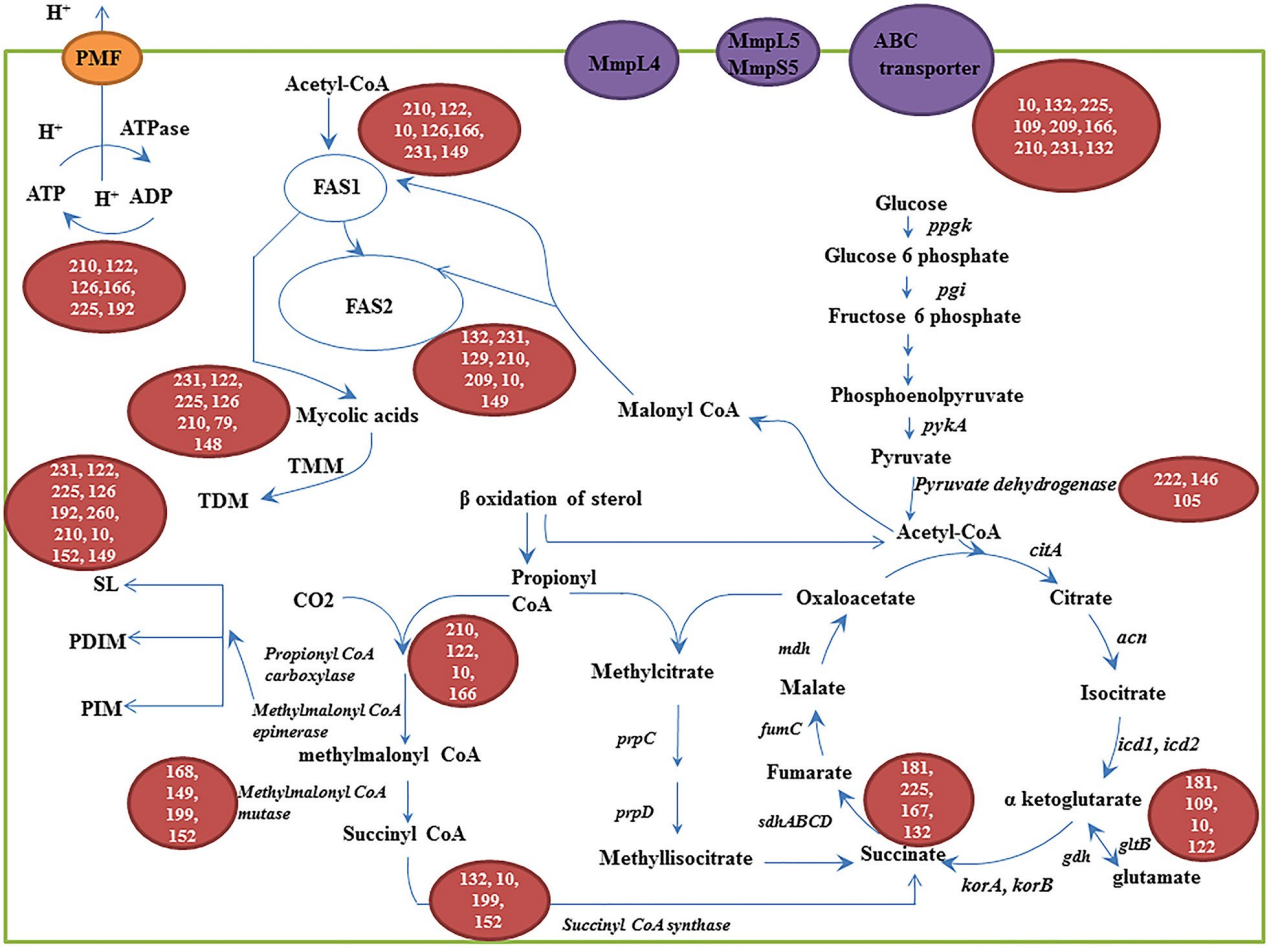
Overview of sRNA candidates associated with the genes involved in various cellular processes in *Mn*-9OHAD/CC. *ppgk* Hexokinase; *pgi* Phosphoglucomutase; *pykA* Pyruvate kinase; *citA* Citrate synthase; *acn* Aconitase; *icd1, icd2* Isocitrate dehydrogenase; *gltB* glutamate synthase; *gdh* glutamate dehydrogenase; *korA, korB* Ketoglutarate ferredoxin Oxidoreductase; *sdhABCD* Succinate dehydrogenase; *fumC* Fumarase; *mdh* Malate dehydrogenase; *prpC* Methylcitrate synthase; *prpD* Methylcitrate dehydratase

Functional enrichment analysis indicated that the target genes of sRNA candidates were preferentially involved in the basic metabolism (such as transport and metabolism of lipid and carbohydrate, and energy catabolism) and the biosynthesis of cell envelope in four groups of *Mn*-CC/C, *Mn*-9OHAD/CC, *Mn*-ADD/CC and *Mn*-BNA/CC. It is suggested that both the basic metabolism and the biosynthesis of cell envelope have close relationships with sterol catabolism in *Mycobacterium* [2, 5]. Acetyl-CoA and propionyl-CoA, two primary metabolites of sterol catabolism, can enter into the basic cellular metabolism and the biosynthesis of cell envelope (Fig. 2) [5, 18]. Therefore, the sRNA candidates related with genes for CCM, lipid metabolism, cell envelope synthesis, transport system, and ATP synthase have been investigated in group *Mn*-9OHAD/CC as a case study.

CCM, which is defined as the enzymatic transformation of carbon through glycolysis, gluconeogenesis, the pentose phosphate shunt and the tricarboxylic acid (TCA) cycle, is very important to the physiology of bacteria [19]. We have found that several sRNA candidates target the genes encoding the key enzymes in CCM, as indicated in Fig. 2 and Additional file 10: Table S8. Pyruvate dehydrogenase complex serves as the metabolic junction between glycolysis and TCA cycle in *Mtb*. Candidates 222 and 146 and candidate 105 are possible regulators for two pyruvate dehydrogenase subunits of *pdhA* and *aceE*, respectively, which were upregulated in *Mn*-9OHAD/CC. For TCA cycle, several sRNA candidates were found to be associated with succinate dehydrogenase, such as candidates 181, 167, 225, and 132 (Fig. 2; Additional file 10: Table S8). In addition, several sRNA candidates were also found to be involved in the regulation of genes encoding for glutamate synthase, glutamate dehydrogenase, such as candidates 181, 109, 10, 101, 174, 122, and 129 (Fig. 2; Additional file 10: Table S8). Propionyl-CoA is a toxic metabolite for mycobacteria, which is a key centered factor leading to the metabolic alterations during sterol catabolism in mycobacteria [20]. There are three different ways to metabolize propionyl-CoA, including the methylcitrate cycle, the vitamin B12-dependent methylmalonyl pathway (MMP), and the incorporation of propionyl-CoA into methyl-branched lipids of cell envelope [21]. Abundant sRNA candidates were found to be related with the key enzyme encoding genes in vitamin B12-dependent methylmalonyl pathway (MMP), including propionyl-CoA carboxylase and methylmalonyl CoA epimerase, as shown in Fig. 2 and Table S8. Generally, the sRNA candidates associated with key enzymes in TCA cycle, glutamate and glutamine synthesis, and MMP were downregulated in *Mn*-9OHAD/CC.

Mycolic acid-arabinogalactan-peptidoglycan layer and a complex set of methyl-branched lipids exposed on the surface of cells, including sulfolipids (SLs), phthiocerol dimycocerosates (PDIM), and phosphatidylinositol mannosides (PIM), are two specific components of the mycobacterial cell envelope, which are synthesized using acetyl-CoA and methylmalonyl CoA as a precursor, respectively [2, 22, 23]. We have found lots of sRNA candidates related with the genes for the biosynthesis of cell envelope (Fig. 2; Additional file 10: Table S8). For mycolic acid, several sRNA candidates are possible regulators for the genes *accA1*, *accD2*, *accD5*, *accD6*, *fas*, *inhA*, *desA1*, *pks13*, and *fbpC*, including candidates 166, 210, 122, 47, 231, 10, 126, 209, 225, and 79. For arabinogalactan, sRNA candidates such as 184, 43, 17, 178, 126, 39, 124, 164, 210, 209, 225, and 10 are related with the genes *glfT2*, *ubiA*, *dprE2*, *embA*, and *embB*. For peptidoglycan, sRNA candidates such as 231, 129, 178, 120, 209, and 169 are possible regulators for the genes *murD*, *pknB*, and *pknG*. In addition, several sRNA candidates were found be possible regulators of the genes for biosynthesis of surface-exposed methyl-branched lipids, including genes *impA*, *rv2611*, and *embC* for PIM, and gene *ppsA* for PDIM (Additional file 10: Table S8). Most sRNA candidates related with the genes for the synthesis of mycolic acid, arabinogalactan, peptidoglycan, SLs, PIM, and PDIM were downregulated in *Mn*-9OHAD/CC.

Some metabolites of sterols, such as AD and ADD, are toxic compounds for mycobacteria cells. The output of these compounds is an important issue for cell survival [2]. In this study, we also identified some sRNA candidates related with efflux pump encoding genes, such as *mmpL4*, *mmpL*5, *mmpS5*, *lfrA*, and *rv1747*. The sRNA candidates involved in efflux pump encoding genes included candidates 124, 10, 225, 109, 209, 167, 166, 210, 231, 70, and so on. The F_1_F_0_-ATP synthase in various prokaryotes is conserved and consists of two regions, i.e. the hydrophobic integral membrane region (F_0_) and the hydrophilic region (F_1_) [24]. Some sRNA candidates involved in F_1_F_0_-ATP synthase subunits, such as *atpD*, *atpG*, *atpFH*, *atpA*, and *atpB*, were also discovered herein (Additional file 10: Table S8), including candidates 17, 169, 166, 122, 225, 210, 128, and so on. The details of sRNA candidates for each efflux pump gene and F_1_F_0_-ATP synthase subunit encoding gene are shown in Additional file 10: Table S8, most of which were downregulated in *Mn*-9OHAD/CC as well.

### Experimental validation of sRNA candidates by qRT-PCR

**Fig. 3:**
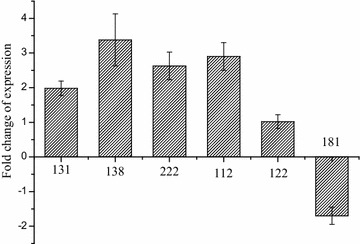
Changes of gene transcription in strain *Mn*-9OHAD compared with strain *Mn*-CC. The values were from three independent cultivations

To evaluate the validity of the above sRNA candidates, six differential expressed sRNA candidates between strains *Mn*-9OHAD and *Mn*-CC, including candidates 131, 138, 112, 181, 222, and 122, which were highly focused in the regulatory networks and related with various key cellular processes, were selected and assessed by qRT-PCR. As shown in Fig. 3, the transcription levels of five candidates, including candidates 131, 138, 222, 112, and 122, were increased in the strain *Mn*-9OHAD. The transcription level of candidate 181 was decreased in the strain *Mn*-9OHAD. Among these results, the transcription levels of candidates 131, 138, 222, 112, and 181 were consistent with the results of transcriptome, but the level of candidate 122 was inconsistent with the results of transcriptome. This inconsistency of candidate 122 between the results of transcriptome and qRT-PCR might be due to two reasons: (1) the operations for strain *Mn*-9OHAD for deep sequencing and qRT-PCR could have a little difference, which might result in the inconsistency; (2) large RNA-seq dataset and bioinformation analysis might generate inaccurate results that could not match the validation experiments.

### Effect of overexpression of candidates 131 and 138 on the production of 9OHAD and growth rates

**Fig. 4:**
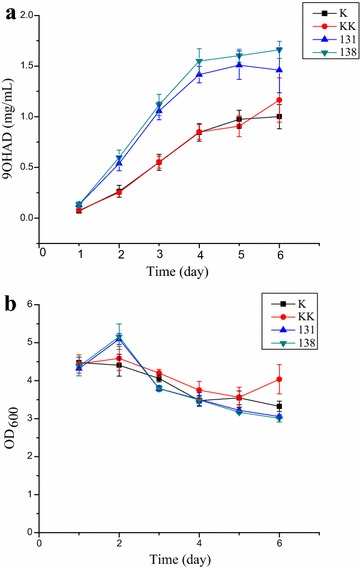
Effect of overexpression of candidates 131 and 138 on strain *Mn*-9OHAD. **a** the production of 9OHAD; **b** the growth rate of cells. K: wide type strain *Mn*-9OHAD; KK: strain *Mn*-9OHAD containing empty vector; 131: strain *Mn*-9OHAD-131 with candidate 131 overexpressed; 138, strain *Mn*-9OHAD-138 with candidate 138 overexpressed

Candidates 131 and 138 are highly focused sRNA candidates in the regulatory networks with upregulation in groups *Mn*-9OHAD/CC, *Mn*-ADD/CC and *Mn*-BNA/CC and downregulation in *Mn*-CC/C. Moreover, they were found to be related with the genes *ltp3*, *fadE26*, *fadE27*, and *fadA5* for sterol catabolism in *Mn*-CC/C, and the expression of which was verified in *Mn*-9OHAD/CC. To explore the functional roles of candidates 131 and 138 in steroid accumulation strains, we engineered their overexpression in strain *Mn*-9OHAD under the control of a strong promoter-*rrnB*, resulting in strains *Mn*-9OHAD-131 and *Mn*-9OHAD-138, respectively. The effect of overexpression on the accumulation of 9OHAD in strains *Mn*-9OHAD-131 and *Mn*-9OHAD-138 were determined, using wide type *Mn*-9OHAD and *Mn*-9OHAD containing empty vector as controls. The time course production of 9OHAD was measured. The introduction of empty vector had little effect on the production of 9OHAD, while the introduction of 131 and 138 led to enhanced production of 9OHAD from 1.5- to 2.3-fold during 6 d’ fermentation, compared with the original strain *Mn*-9OHAD (Fig. 4a). The maximal increasement was observed at 2 and 3 d. At the late stage of fermentation, the enhancement was unobvious, which might be attributed to the potential toxic effect of steroid intermediates and the consumption of sterols. The effect of overrepresentation of candidates 131 and 138 on the growth rates of cells was also explored, as shown in Fig. 4b. The introduction of empty vector had little effect on the growth rate of cells (Fig. 4b). At the initial stage of fermentation, the growth rate of cells showed a little enhancement in strains *Mn*-9OHAD-131 and *Mn*-9OHAD-138, compared with the control strain *Mn*-9OHAD (Fig. 4b). During the late stage of fermentation, the growth rate of cells in strains *Mn*-9OHAD-131 and *Mn*-9OHAD-138 was comparable or a little decreased than that of strain *Mn*-9OHAD (Fig. 4b). In summary, the overexpression of candidates 131 and 138 showed little effect on the growth rate of cells.

### Functional characterization of target genes of candidates 131 and 138

**Fig. 5:**
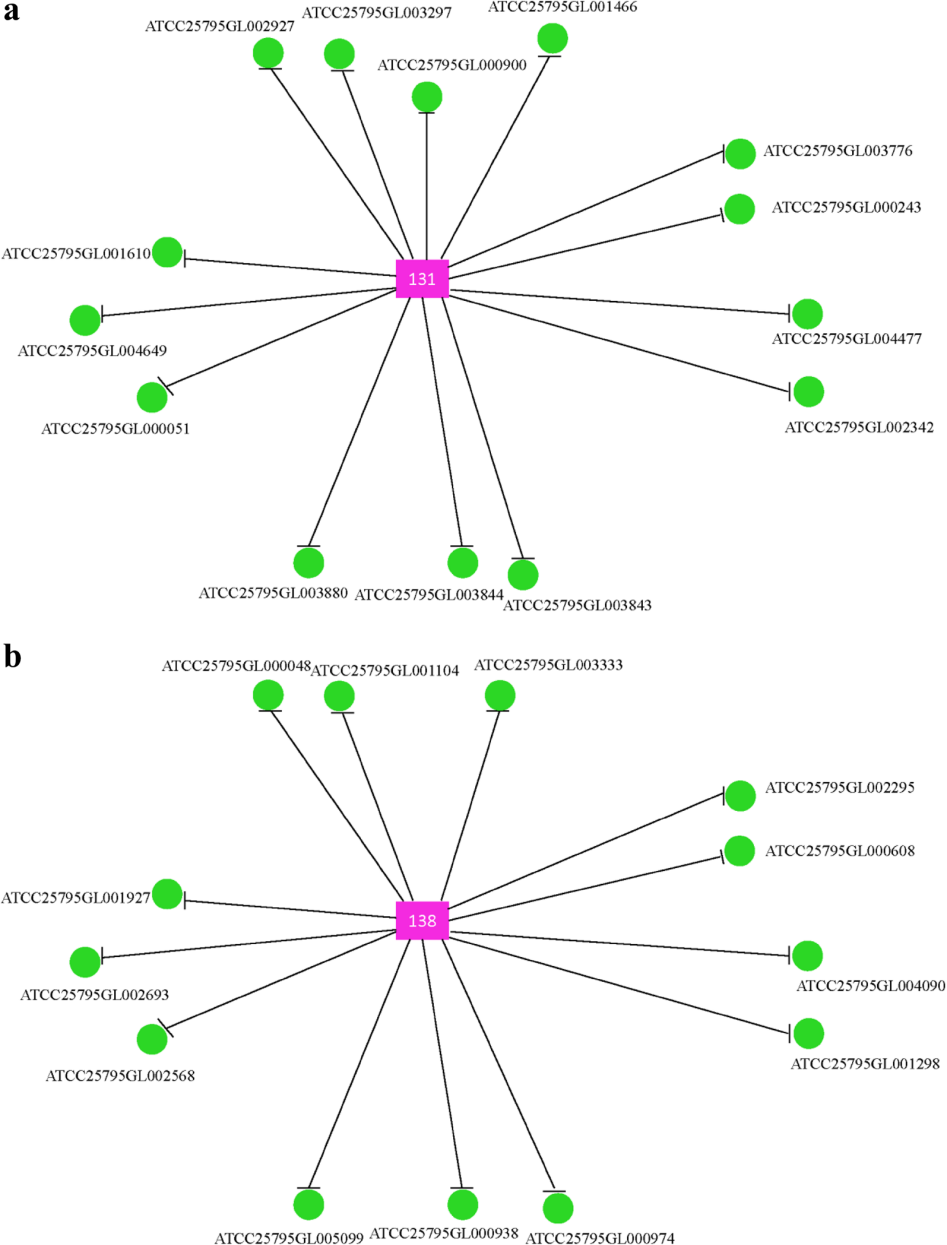
Potential target genes of candidate 131 (**a**) and 138 (**b**)

To explore the putative reason for enhanced production of 9OHAD in *Mn*-9OHAD-131 and *Mn*-9OHAD-138, the potential target genes of 131 and 138 were analyzed in group *Mn*-9OHAD/CC. As shown in Fig. 5, a total of 14 and 13 potential target genes were found for candidates 131 and 138, respectively. COG analysis and functional characterization indicated the target genes of candidate 131 were involved in transcription, inorganic ion transport and metabolism, amino acid transport and metabolism, carbohydrate transport and metabolism, energy production and conversion, and signal transduction (Additional file 11: Table S9). Additionally, for candidate 138, COG analysis and functional characterization indicated that the target genes were involved in transcription, cell motility, lipid transport and metabolism, amino acid transport and metabolism (Additional file 11: Table S9).

## Discussion

The aim of this work was to uncover non-coding sRNA system through transcriptome analysis in wide type *Mn* and 9OHAD, ADD, and BNA producing *Mn* strains. Application of deep sequencing to study the transcriptional landscape of *Mn* has uncovered an abundance of non-coding sRNA candidates in the work. Modulation of gene expression through small non-coding RNAs is one of the most extensively studied classes of regulators in bacteria, which can modulate transcription, translation, mRNA stability, and DNA maintenance and play a key role in cellular physiology of bacteria [9]. Thus, uncovering and characterization of these non-coding sRNA candidates is imperative for understanding the complex biology underlying sterol metabolism and steroid accumulation in *Mn* cells.

Facilitated with high throughput sequencing, we have discovered novel transcripts located in intergenic regions according to the method described in 2008 [25]. The novel transcripts that could not blast against NR databases were considered to be sRNA candidates in the work. A total of 263 sRNA candidates have been predicted herein, which vary in length and location (Table S2). The typical size of bacterial sRNAs varies from 50 to 500 nucleotides [9]. The length of sRNA candidates in *Mn* cells ranges from 200 to 500 bp, the distribution of which is broadly accord with the generally reported length of bacterial sRNAs. The location of sRNA candidates suggests they may function to prevent the translation of target mRNAs by steric hindrance of the ribosome binding site. Moreover, the secondary structure of sRNA candidates was investigated in the work (Additional file 2: Table S2), which facilitated the understanding of functions of sRNAs in the sterol catabolism and steroid accumulation. The annotation of sRNA candidates was mostly obtained using the database of Rfam based on consensus secondary structure, and the annotation results were abundant and complex (Additional file 2: Table S2). To study the function of sRNA candidates, further investigations need to be considered.

RPKM values were used to quantify the transcript levels of sRNA candidates and their target genes, which facilitated transparent comparison of transcript levels between different samples [11, 25]. RPKM values of sRNA candidates for samples *Mn*-C, *Mn*-CC, *Mn*-9OHAD, *Mn*-ADD, and *Mn*-BNA, together with comparative results in groups *Mn*-CC/C, *Mn*-9OHAD/CC, *Mn*-ADD/CC and *Mn*-BNA/CC were investigated in the work. Finally, multiple differentially expressed sRNA candidates were detected in *Mn*-CC/C, *Mn*-9OHAD/CC, *Mn*-ADD/CC and *Mn*-BNA/CC. The differential expression under specific environmental stresses suggests their relevance to sterol catabolism and steroid accumulation in *Mn*. The number of sRNA candidates showing significantly differential expression in groups *Mn*-CC/C, *Mn*-9OHAD/CC, *Mn*-ADD/CC and *Mn*-BNA/CC was comparable. Interestingly, more downregulated sRNAs were found in groups *Mn*-9OHAD/CC, *Mn*-ADD/CC and *Mn*-BNA/CC than that of *Mn*-CC/C. Considering the dominating negative interactions between sRNAs and their targets [26], it was speculated that more genes for sterol catabolism and intermediate production with direct and/or indirect interactions could be activated in steroid producing strains. In addition, many sRNA candidates were found to be prevalently upregulated or downregulated in strains *Mn*-9OHAD, *Mn*-ADD and *Mn*-BNA. It was suggested that these sRNAs might function in the regulation of the biotransformation of sterols to accumulate key steroid intermediates. The sRNA candidates, preferentially expressed at specific stages or prevalently expressed at various strains, may raise interesting future work to elucidate the detailed roles of these sRNA candidates in global metabolic regulation of sterol catabolism to produce valuable steroid intermediates.

Given the abundance of sRNAs of unknown function, it is important to identify their cellular targets. A few sRNAs regulate target genes by binding to regulatory proteins, but most sRNAs of known function regulate gene expression by base pairing to specific mRNAs, which often occurs in the immediate vicinity of, or overlapping with, translation initiation sites on the target mRNAs with short and imperfect complementarity [12, 26]. Thus, one frequently observed mechanism for regulating gene expression by sRNAs involves the competition between sRNAs and initiating ribosomes that results in translational inhibition [9, 26, 27]. Based on this mechanism, we have predicted the target genes of sRNA candidates incorporating target site accessibility and seed regions using an approach of IntaRNA, where a combined energy score of the interaction was calculated as the sum of the free energy of hybridization and the free energy required for making the interaction sites accessible [26, 27]. As shown in Additional file 5: Table S4, one sRNA candidate could regulate multiple target genes, while one mRNA could be regulated by several sRNAs. The regulative relations between sRNA candidates and target genes were very complicated, with 15519, 16583, 14217, and 15,274 sRNA-target relationships found in groups *Mn*-CC/C, *Mn*-9OHAD/CC, *Mn*-ADD/CC and *Mn*-BNA/CC, respectively. Thus, it was difficult to investigate the target genes and functional roles of sRNA candidates one by one. To investigate the functional roles of target genes that these sRNA candidates might regulate, we identified molecular functions and pathways enriched in the sRNA-targeted genes in four groups of *Mn*-CC/C, *Mn*-9OHAD/CC, *Mn*-ADD/CC and *Mn*-BNA/**C**C. As shown in Additional file 8: Table S6, an initial analysis of sRNA target pathways indicated that the sRNA candidates preferentially targeted genes involved in the basic metabolism, lipid transport and metabolism, and cell envelope components. This preliminary finding suggested that the expression of sRNAs might be not only as part of the stress response for basic metabolism, but was actively involved in the regulation of other critical functions such as lipid transport and metabolism, and cell envelope components.

Now, small RNAs have been accepted to be a kind of universal and fundamental factors involving in the physiology and metabolism of organisms. Thus, characterization of the functions of sRNAs is becoming more important to better understand the complex phenotype of specific organism [28]. Because of the complex relationships between sRNAs and their targets, we were interested in exploring the possibility to develop regulatory networks between sRNA candidates and their target mRNAs to further elucidate the functional roles of sRNA candidates. As shown in Additional file 6: Figure S2, four regulatory networks were constructed in groups *Mn*-CC/C, *Mn*-9OHAD/CC, *Mn*-ADD/CC and *Mn*-BNA/CC using Cytoscape based on the prediction of sRNAs and targets, respectively. Several upregulated and downregulated sRNA candidates with focused roles were found in these regulatory networks, which led us to concentrate on these focused candidates. Accordingly, we explored the most abundant upregulated and downregulated sRNA candidates with focused roles in the regulatory networks of *Mn*-9OHAD/CC, *Mn*-ADD/CC and *Mn*-BNA/CC. Several highly focused sRNA candidates were discovered to be prevalently existed in networks of *Mn*-9OHAD/CC, *Mn*-ADD/CC and *Mn*-BNA/CC, including upregulated candidates 131, 138, 222, 79, and 112 and downregulated candidates 225, 209, 122, 210, 166, 152, 164, 70, and 97. It was assumed that these prevalently existing sRNAs in *Mn*-9OHAD/CC, *Mn*-ADD/CC and *Mn*-BNA/CC could contribute to the production of key steroid intermediates, such as 9OHAD, ADD, and BNA. These regulatory networks also indicated that regulation of the associated cellular processes, such as lipid transport and metabolism, amino acid transport and metabolism, signal transduction, and cell envelope biosynthesis, might involve a complex interplay between sRNA candidates and their target genes, as shown the details in Additional file 7: Table S5. The networks could uncover direct and/or indirect sRNA-target interactions to a certain extent, and could be used to elucidate functions and regulatory roles of sRNA candidates [28].

Microbial steroid transformation has attracted considerable attention for the generation of novel steroidal drugs, as well as for producing bioactive steroid intermediates from low-cost sterols [1]. In a breakthrough study, van der Geize et al. identified a complete suite of 51 genes required for sterol catabolism in *R. jostii*, which are likewise found within an 82-gene cluster in *Mtb* [6, 18]. We investigated the putative sRNA candidates associated with genes responsible for sterol catabolism, as shown in Table 6. It was found that the sRNA candidates for the regulation of genes in sterol catabolism operon were generally different but had shared features in groups *Mn*-CC/C, *Mn*-9OHAD/CC, *Mn*-ADD/CC and *Mn*-BNA/CC. Moreover, several highly focused sRNA candidates in the regulatory networks were found to be regulators for the genes in sterol catabolism operon, such as 79, 209, 225, 210, 131, and 138. To utilize sterol substrates, the *Mn* cells needed the upregulated genes for sterol catabolism, which could be regulated by these downregulated sRNA candidates, indicating an important role of sRNAs in sterol catabolism.

The regulation of sRNA candidates for CCM, cell envelope biosynthesis, transporters, and F_1_F_0_-ATP synthase complex for ATP synthesis in group *Mn*-9OHAD/CC was investigated herein. Low productivity of steroid intermediates from sterols remains the bottleneck for commercial use of most sterol-transforming mycobacteria. Usually, low efficiency in the sterol biotransformation process is partly ascribed to two inherent problems: the low water solubility of hydrophobic sterols (usually less than 1 μM) and the potentially toxic final products to the microbial cells [1, 2]. As well-known mycolate-rich bacteria, mycobacteria have exceptionally thick and hydrophobic cell envelope [22]. The hydro-phobicity may allow the cells to adhere to hydrophobic substrates when the cells need sterol as nutrient at the initial stage of fermentation, but the unusual thickness may also form a barrier against the entry of exogenous sterols into the cell when the toxic steroid product affects the cells at the later stage of fermentation [2]. We proposed that some sRNA candidates participated in the biosynthesis of cell envelope might function in the regulation process to response to environmental variations in *Mn* cells. Most sRNA candidates related with the genes for synthesis of cell envelope were downregulated in *Mn*-9OHAD/CC. It was assumed that these upregulated genes regulated by sRNAs were responsible for the biosynthesis of hydrophobic cell envelope to facilitate the uptake of sterol substrate at the initial stage of fermentation. The cellular toxicity of steroids to microbial cells is potentially one of the major factors limiting the productivity of steroid intermediates from sterol transformation [1, 2]. In *Mtb*, the complex multiplicity of efflux pumps illustrates the ability of *Mtb* to transport a variety of toxic compounds or antibiotics, a key feature of which is their dependence on the proton motive force or the availability of ATP, linking drug efflux to energy metabolism and the electron transport chain [24]. We investigated the sRNA candidates involving in the genes encoding some well-known efflux pumps, such as *mmpL4*, *mmpL*5, *mmpS5*, *lfrA*, and *rv1747*, as well as sRNA candidates for ATP synthesis. Most sRNAs for regulating the genes encoding for efflux pump and F_1_F_0_-ATP synthase subunit were also downregulated, indicating these upregulated target genes might be related with the output of toxic steroid intermediates. Among these candidates, it was worth noting that some sRNAs, such as candidates 225, 210, 109, 10, 122 and 166, were possible prevalent regulators for various aspects, including the CCM, cell envelope biosynthesis, efflux pumps, and ATP synthesis in *Mn*. It was assumed that these processes could be connected through the global regulation functions of some sRNA candidates to a certain extent.

To explore the functional role of sRNA candidates in *Mn* cells, we manipulated the overexpression of candidates 131 and 138 in strain *Mn*-9OHAD. Candidates 131 and 138 were highly focused sRNA candidates in the regulatory networks of *Mn*-CC/C, *Mn*-9OHAD/CC, *Mn*-ADD/CC and *Mn*-BNA/CC, which were also related with the sterol catabolism genes of *ltp3*, *fadE26*, *fadE27*, and *fadA5* in *Mn*-CC/C. The overexpression of candidates 131 and 138 in strain *Mn*-9OHAD caused enhanced production of 9OHAD from 1.5- to 2.3-fold during 6 d’ fermentation and a little enhanced growth at the initial stage and a slight reduced growth at the late stage of fermentation. Functional characterization of target genes of candidates 131 and 138 indicated that the targets were involved in various aspects in cellular process, including transcription, lipid transport and metabolism, energy production and conversion, signal transduction, cell motility, inorganic ion transport and metabolism, amino acid transport and metabolism, and carbohydrate transport and metabolism. It was speculated that the candidates 131 and 138 might influence the production of 9OHAD by direct and/or indirect connections with sterol catabolism through various cellular processes. However, the exact regulation mechanism underlying candidates 131 and 138 and their respective targets need to be explored in the future work.

## Conclusions

RNA sequencing provides a new perspective on the genome of *Mn* by revealing an extensive presence of intergenic sRNAs. In the present work, we performed massive sequencing on the Illumina platform to obtain a comprehensive description of noncoding sRNA candidates in *Mn* cells for the catabolism of sterol and production of key steroid intermediates of 9OHAD, ADD, and BNA. The results could imply the involvement of these intergenic sRNAs in the adaptation of mycobacteria to the environmental variations and uncover a wide range of non-coding sRNA candidates with the potential to influence the patterns of gene expression during production of steroids. By constructing regulatory networks in groups *Mn*-CC/C, *Mn*-9OHAD/CC, *Mn*-ADD/CC and *Mn*-BNA/CC based on the interactions of sRNAs and targets, the comprehensive regulatory roles of sRNA candidates were uncovered in various cellular processes of *Mn*. Several highly focused sRNA candidates, who were discovered to be prevalently existed in regulatory networks, played important roles in the sterol catabolism, CCM, cell envelope biosynthesis, efflux pumps, and ATP synthesis. Further targeted analysis by overexpression or deletion of them will provide insights into the molecular mechanisms of these sRNA candidates and open a broad range of opportunities in the field.

## Methods

### Strains and culture conditions

In the present work, we have conducted RNA sequencing to study the noncoding transcriptome of five samples, including *Mn*-C, *Mn*-CC, *Mn*-9OHAD, *Mn*-ADD, and *Mn*-BNA. Strain *Mn*-9OHAD was constructed by deletion of a 3-ketosteroid-Δ1-dehydrogenase encoding gene (*kstd*1) in wild type *Mn* ATCC25795, which could produce 9OHAD. Strain *Mn*-ADD was constructed by deletion of a 3-ketosteriod-9α-hydroxylase encoding gene (*kshA1*) in *Mn* ATCC25795, which could produce ADD. Strain *Mn*-BNA was constructed by deletion of a β-hydroxy-acyl-CoA dehydrogenase encoding gene (*hsd4A*) in *Mn*-ADD, which could produce 1,4-BNA. The mutant strains of *Mn*-9OHAD, *Mn*-ADD, and *Mn*-BNA were constructed by deletion of specific genes as described in previous work [4, 7]. These strains were aerobically cultured in MYC/01 medium as described previously [7]. For steroid biotransformation, 10.0 g/l glucose and 0.5 g/l phytosterol emulsified in Tween 80 (at 120 °C for 1 h) were supplemented into MYC/01, which was called MYC/02 medium. The final concentration of Tween 80 was no more than 0.08 % in the MYC/02 medium. The samples for strains *Mn*-C, *Mn*-CC, *Mn*-9OHAD, *Mn*-ADD, and *Mn*-BNA were taken at 48 h after fermentation.

### RNA isolation, preparation of cDNA library and sequencing

Total RNA of samples *Mn*-C, *Mn*-CC, *Mn*-9OHAD, *Mn*-ADD, and *Mn*-BNA were isolated using the FastRNA™ Pro Blue Kit and the Fast Prep-24™ Instrument (MP Biomedicals, SantaAna, CA). NanoDrop (NanoDrop Technologies,Wilmington, DE) was used to verify the concentration and quality of each RNA sample. Total RNA was incubated with Rnasefree DNase Set (Qiagen) to remove DNA contamination according to the manufacturer’s instructions. Messenger RNA (mRNA) was further purified by depleting ribosomal RNA (rRNA) and tRNA with Terminator™ 5′-phosphate dependent exonuclease (Epicenter, Madison, WI) digestion. Double-stranded cDNA was synthesized using a kit obtained from Beijing Dingguo Chang sheng Biotechnology Co., Ltd., (Beijing, China). RNA sequencing was conducted with an Illumina Genome Analyzer IIx according to the manufacturers’ protocols.

### Prediction of sRNA candidates in silico

The overview for analysis of sRNA candidates is shown in Fig. 1. We selected gene models with length ≥100 bp and average depth ≥2, and then selected novel transcripts located in the intergenic regions (200 bp far from 3′ of upstream gene and 200 bp far from 5′ of downstream gene) among these gene models according to the method described in 2008 [25]. Blast the novel transcripts against non-redundant (NR) databases and the novel transcripts that could not blast against NR databases were considered as sRNA candidates.

To explore the relevance of sRNA expression to sterol catabolism and steroid accumulation in *Mn* cells, differential expression of sRNA candidates was explored in the wide type *Mn* with vs without sterol addition (named *Mn*-CC/C), and the steroid intermediate producing *Mn* strains vs wide type *Mn* with sterol addition (named *Mn*-9OHAD/CC, *Mn*-ADD/CC and *Mn*-BNA/CC), respectively. Reads per Kilobase per Million mapped reads (RPKM) was used to measure the relative expression of sRNA candidates and their target genes in the work, which reflected the molar concentration of a transcript in the starting sample by normalizing the number of reads mapping to a gene to both the feature lengths and the total number of reads [11, 25]. The differentially expressed sRNAs were selected using the following criteria: (1) Fold change >2 or <0.5, i.e. log_2_FC >1 or <−1; (2) *P* value <0.05, and FDR <0.05.

For the annotation of candidate sRNAs, two methods were used as follows: (1) we searched databases of sRNAMap (http://srnamap.mbc.nctu.edu.tw/download.php), sRNATarBase (http://ccb.bmi.ac.cn/srnatarbase/download.php) and SIPHI (http://newbio.cs.wisc.edu/sRNA/published_annotations/) based on the similarity of sequences using Blast (e value <0.00001); (2) we searched the database of Rfam (http://rfam.janelia.org/) based on the consensus secondary structure. Secondary structures of sRNA candidates were predicted using the Vienna RNA package. This server (http://rna.tbi.univie.ac.at/) provides programs, web services, and databases, which are related to our work on RNA secondary structures.

### Target prediction of sRNA candidates

IntaRNA (http://rna.informatik.uni-freiburg.de:8080/IntaRNA.jsp) was used as a tool to predict the gene targets of differential expressed sRNAs as described previously [26, 27]. The scoring was based on the hybridization free energy and accessibility of the interaction sites in both molecules. The interaction contained an interaction seed, i.e. a region of (nearly) perfect sequence complementarity to facilitate interaction initiation. Accessibility was defined as the free energy required unfolding the interaction site in each molecule. For the calculation of these unfolding energies, global folding of the sRNA candidates and local folding of the mRNA were assumed.

### Construction of sRNA-target regulatory networks

The sRNA-target regulatory networks were generated by connecting gene nodes and differently expressed sRNAs to represent direct and/or indirect biological relationships using the open source software Cytoscape (Cytoscape Consortium, San Diego, CA, USA) [29].

### Quantitative reverse transcription-PCR (qRT-PCR)

Total RNA extraction, quantitation, verification, removal of DNA and cDNA synthesis were performed as described in section “RNA isolation, preparation of cDNA library and sequencing”. QRT-PCR analyses of cDNA samples were performed on the StepOneTM Real-Time PCR System (Applied Biosystems) using a qRT-PCR (TER010-2) kit from Beijing Dingguo Chang sheng Biotechnology Co.,Ltd.,(Beijing,China). The primers for qRT-PCR (Table 7) were synthesized in Genscript (Nanjing, China). Details of reaction system and condition were conducted as described previously [4].

**Table 7:** Primers used in the qRT-PCR

SRNA ID	Product length (bp)	Sense Primer (5′–3′)	Anti-sense primer (5′–3′)
131	202	CTACCGCCCCGAAAGTCT	AGGACAGACAAGGCAACAGC
138	150	CTCGTTCTGCTTCTCGTC	ACATACGGCTGGAACATC
112	180	CTGTTCGGCCAGGGTCAG	GAATTCGTGGTCTCCGTCAC
181	146	CTTCCTCCAACCCTTCAC	GATTCGACACCCCATACC
222	55	TGTACTCCTCGTCGGTGA	TTGCAGGAGTTGGAGACA
122	112	GTGGAAGACCCCACTGAC	GGTGCGACTACTGGTGAA

### Plasmid and strain construction for overexpression of candidates 131 and 138

We constructed the overexpression plasmid for sRNAs by replacing the *Xba*I-*Hind*III fragment containing the Hsp60 promoter in plasmid pMV261with the *Xba*I-*Hind*III fragment spanning −200 to −8 of the *rrnB* promoter from *M. smegmatis* [30]. In this overexpression vector, the sRNAs were inserted as a *Hind*III fragment downstream of the −10 region, and such that sRNA candidates could be expressed from their own transcription start site. A synthetic transcriptional terminator (CCCCGCGAAAGCGGGGTTTTTTTTTT) was inserted at the *Hind*III site downstream of the sRNA 3′ end to ensure the efficiency of termination. A gene fragment containing −200 to −8 of the *rrnB* promoter from *M. smegmatis*, a *Hind*III site downstream of −10 region, and a synthetic transcriptional terminator downstream *Hind*III site and flanking *Xba*I and *Hap*I was synthesized in Genscript (Nanjing, China). This fragment was digested with *Xba*I and *Hpa*I, and then ligated into pMV261 digested with the same enzymes to construct the overexpression plasmid of pMV261-*rrnB*. The candidates 131 and 138 were inserted into pMV261-*rrnB* using primers 5′-TATCAAGCTTTCTCGCTACCGCCCCGAAAGT-3′ (F-131) and 5′-TATCAAGCTTCGACTGTGGCCGAGGACAGACA-3′ (R-131) and 5′-TATCAAGCTTGTGCCCATCAGACTAGAGG-3′ (F-138) and 5′-TATCAAGCTTGAAGCCGATGGCGGAGTT-3′ (R-138), creating recombinant plasmids pMV261-*rrnB*-131 and pMV261-*rrnB*-138, respectively. The primers were synthesized in Genscript (Nanjing, China). The recombinant plasmids were transformed into *Escherichia coli* DH5α using a heat shock method at 42 °C for 90 s. Subsequently, the correct recombinant plasmids from *E. coli* DH5α were verified using DNA sequencing and transferred into strain *Mn*-9OHAD by electroporation, creating strains *Mn*-9OHAD-131 and *Mn*-9OHAD-138, respectively.

### Steroid analysis and determination of optical density (OD)

The 9OHAD was analyzed by HPLC using reversed-phase C18-column (250 mm × 4.6 mm) (Agilent, USA) at UV absorption of 254 nm. Mobile phase composed of methanol: water (80: 20, v/v) was used. Standard substance was used as positive control. To determine the growth curves of cells, the OD was determined at 600 nm in duplicate using a Hitachi U-1100 spectrophotometer. The OD values of wide type *Mn*-9OHAD, strain *Mn*-9OHAD containing empty vector, *Mn*-9OHAD-131 and *Mn*-9OHAD-138 were measured at 1, 2, 3, 4, 5, and 6 d, respectively.

## Additional files

10.1186/s12934-016-0462-2 Novel transcripts found in strains *Mn*-C, *Mn*-CC, *Mn*-9OHAD, *Mn*-ADD, and *Mn*-BNA.
10.1186/s12934-016-0462-2 Details of sRNA candidates, including location in chromosome, upstream and downstream genes, sequences, secondary structures, alignment results with known database (Rfam).
10.1186/s12934-016-0462-2 Length distribution of all sRNA candidates.
10.1186/s12934-016-0462-2 All RPKM values of sRNA candidates in strains *Mn*-C, *Mn*-CC, *Mn*-9OHAD, *Mn*-ADD, and *Mn*-BNA and the differential expression of sRNA candidates in comparable groups *Mn*-CC/C, *Mn*-9OHAD/CC, *Mn*-ADD/CC, and *Mn*-BNA/CC.
10.1186/s12934-016-0462-2 Target genes of sRNA candidates in comparable groups *Mn*-CC/C, *Mn*-9OHAD/CC, *Mn*-ADD/CC, and *Mn*-BNA/CC.
10.1186/s12934-016-0462-2 Negative networks of differentially expressed sRNA candidates and their target genes. (a): *Mn*-CC/C; (b): *Mn*-9OHAD/CC; (c) *Mn*-ADD/CC; and (d): *Mn*-BNA/CC. Squares represent the upregulated (amaranth) or downregulated sRNA candidates (blue); circles represent the putative upregulated (red) or downregulated (green) target genes; links represent the regulation of sRNAs on their target genes.
10.1186/s12934-016-0462-2 Negative analysis results of sRNA candidates and target genes in comparable groups *Mn*-CC/C, *Mn*-9OHAD/CC, *Mn*-ADD/CC, and *Mn*-BNA/CC.
10.1186/s12934-016-0462-2 COG analysis of target genes of sRNA candidates in comparable groups *Mn*-CC/C, *Mn*-9OHAD/CC, *Mn*-ADD/CC, and *Mn*-BNA/CC.
10.1186/s12934-016-0462-2 Details of sRNA candidates associated with sterol degradation genes in comparable groups *Mn*-CC/C, *Mn*-9OHAD/CC, *Mn*-ADD/CC, and *Mn*-BNA/CC
10.1186/s12934-016-0462-2 Details of sRNA candidates associated with the genes involved in basic metabolism, cell envelope biosynthesis, efflux pumps, and ATP synthesis in group *Mn*-9OHAD/CC.
10.1186/s12934-016-0462-2 Details of functional characterization of potential target genes of sRNA candidates 131 and 138.
