# Supplementary figures and images for: RNA-Seq analysis uncovers non-coding small RNA system of Mycobacterium neoaurum in the metabolism of sterols to accumulate steroid intermediates
Source: Microb Cell Fact. 2016 Apr 25;15:64. doi: 10.1186/s12934-016-0462-2 (PMC4845491; doi:10.1186/s12934-016-0462-2)

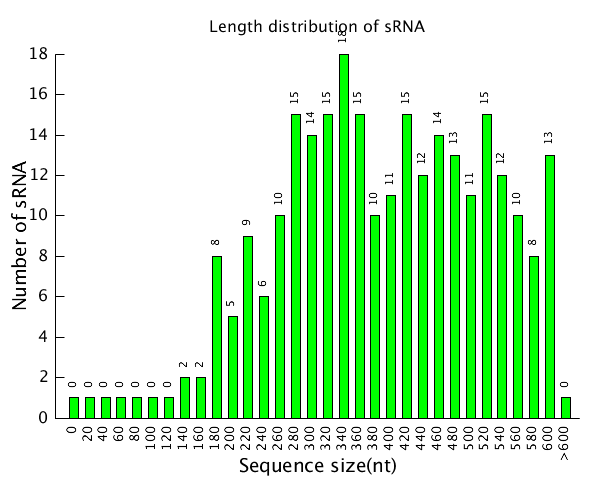

Supplement: Supplementary file 3 — 10.1186/s12934-016-0462-2 Length distribution of all sRNA candidates. [file 12934_2016_462_MOESM3_ESM.png]

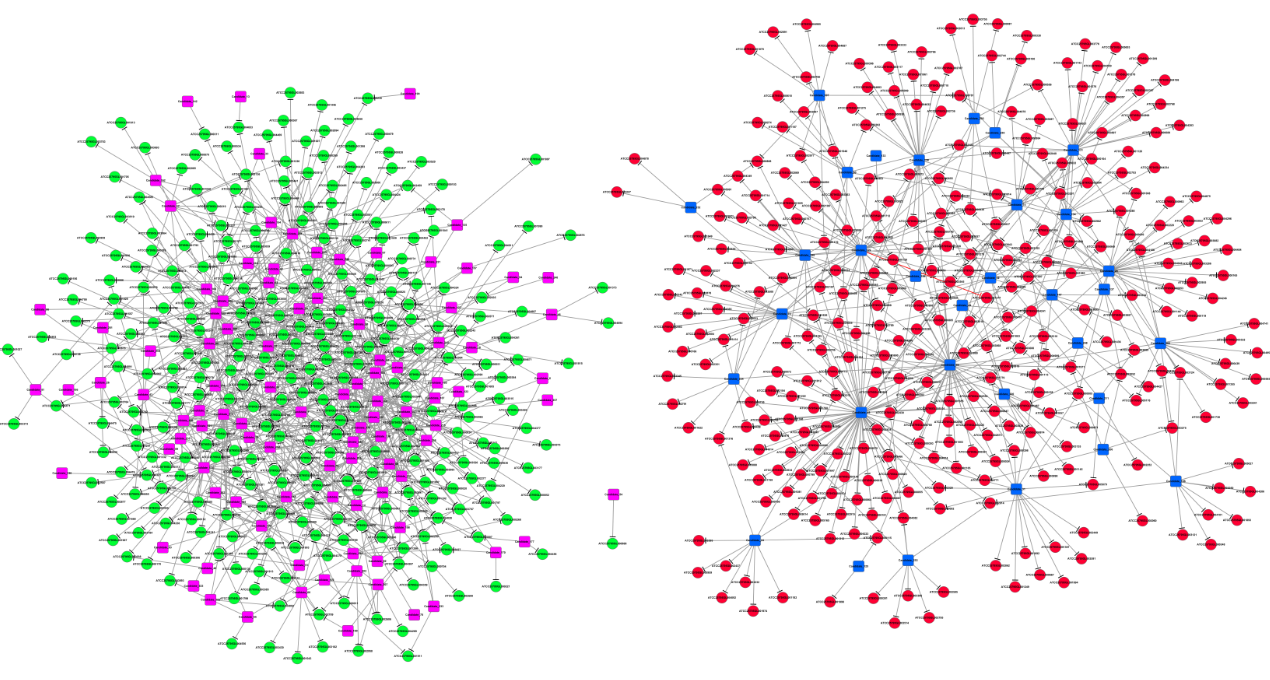


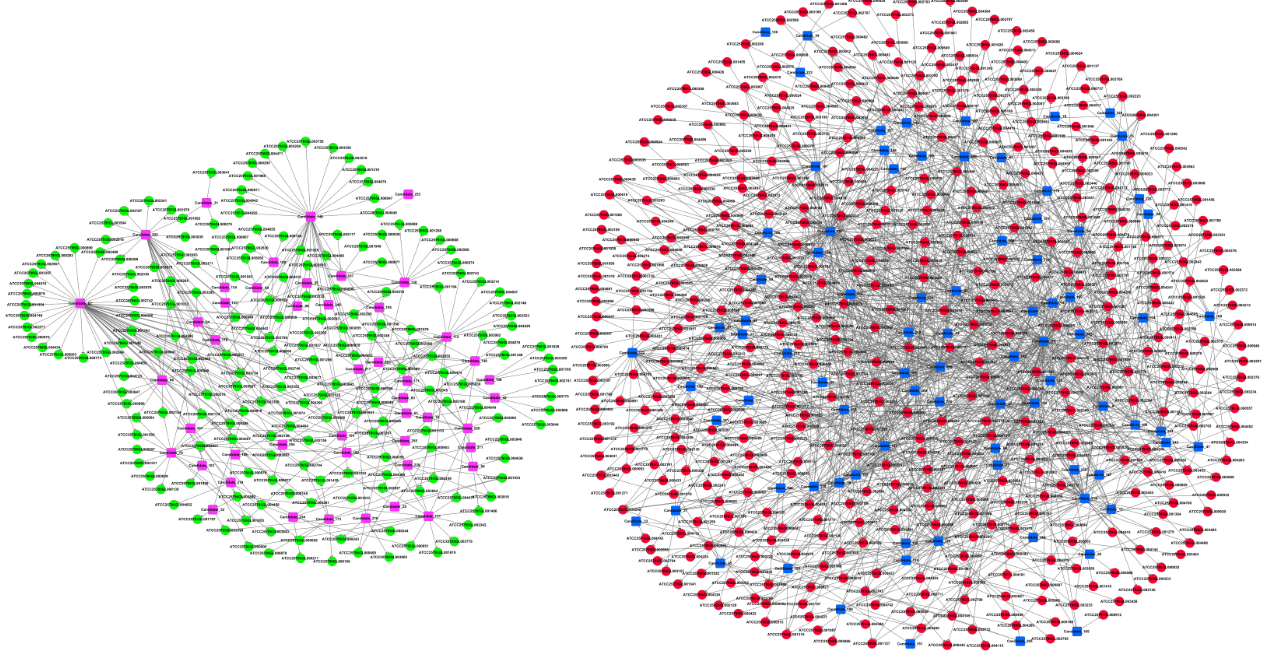


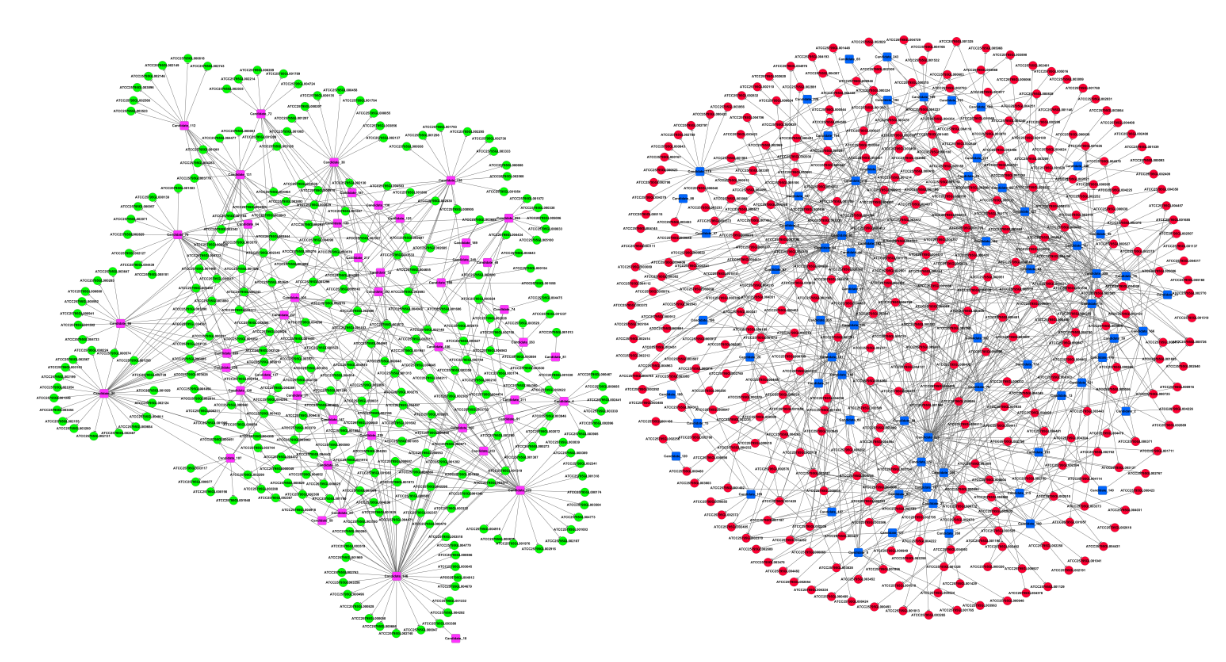


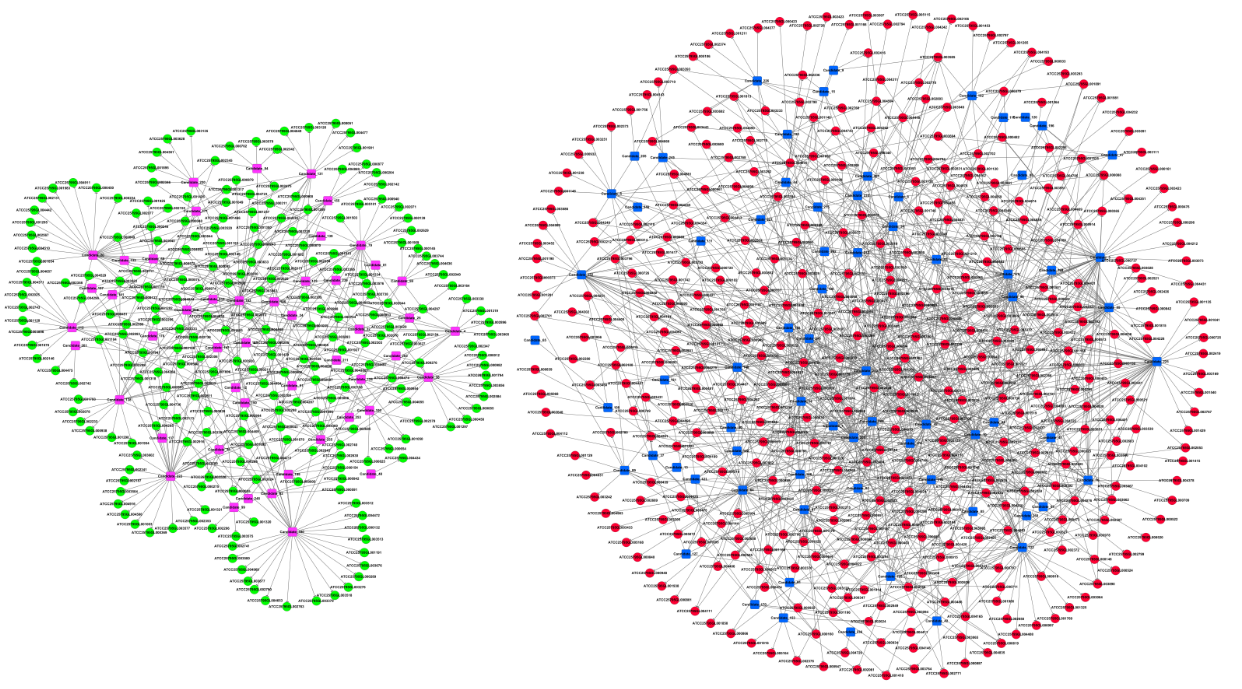

Supplement: Supplementary file 6 — 10.1186/s12934-016-0462-2 Negative networks of differentially expressed sRNA candidates and their target genes. (a): Mn-CC/C; (b): Mn-9OHAD/CC; (c) Mn-ADD/CC; and (d): Mn-BNA/CC. Squares represent the upregulated (amaranth) or downregulated sRNA candidates (blue); circles represent the putative upregulated (red) or downregulated (green) target genes; links represent the regulation of sRNAs on their target genes. [file 12934_2016_462_MOESM6_ESM.docx]
